# Dr. Howard B. Besemer

**Published:** 1918-04

**Authors:** 


**Dr. Howard B. Besemer,** New York University, 1891 and Cleveland Hom. Med. College, 1892, died in Ithaca Feb. 8, age 48.

